# The Moral Basis of Private Property.—I

**Published:** 1890-02-22

**Authors:** 


### The Moral Basis of Private Property.

By the Bishop of London.

### I.—PRIVATE PROPERTY.

The Bishop of London has recently addressed his clergy on Socialism, which should be studied, he says, although it is not always advocated in its extreme form. Socialism in its extreme form, says the bishop, asserts that the private ownership of property is a bad and a wrong thing, and ought not to exist. Why was it, then, that man, as man, could not do without the principle and practice of the private ownership of property. That was the question. If man had no relation to anyone except to the community, there might be nothing more to be said; but the moment you introduce the idea of the family, the home, and domestic life, private property came in with great force, and there was no escape from the principle. Domestic life meant that one had obligations to certain human beings, which he had not to others, that he was bound by closer ties to some than to others; it meant a distinct preference for some over others, a different duty towards them. All the various safeguards which protected marriage marked the distinctions between man and the beasts. The lower appetite, the sexual appetite, which man shared with the beasts was only possible for a moral being under the restraints of domestic life. The possibility there could be the spiritual in man, and that he should be therefore capable of the highest moral life, and yet that he should live in a certain way as an animal—this possibility could not be admitted except on the condition that he lived under the restraints and safeguards of the home. If there was to be amongst human beings this inner circle, this different relation to each other from the relation to all outside, there must obviously be a place where those so bound to each other might live together, and there inevitably followed the duty of the members of that inner circle to provide for one another. The sustenance of those who lived in the home became the duty of the man to whom the home belonged. Provision for the comfort of every member of the family came under the rule of the same duty, and this could not be done apart from the principle of private property. And it was in this that the moral basis of private property would always be found.

It had been urged that the moral basis of private property was to be found in a man's labour. A man, it was said, had no private property in things which exist. God had created them, and they were for the whole race; but a man's labour was his own. But the Socialists had a complete answer to this; what business, they said, has a man to put his labour into any particular thing? A man carved a stick, for instance, and said he owned it because he had carved it. But, it was asked, "What right had you to the stick? you stole it to begin with; it belongs to the community, and you ought to have waited for leave." These objections did not apply to domestic life as giving the moral basis for private property, for that life was inherent in human nature. When God created the family, he created the means of maintaining it, and the duty of the members to supply those means.

But then, it might be said—provide for the needs of the family, and no more. This led to the reflection that, taking climate, population, &c., into account, there were places where the available food would not nourish people the whole year round. Hence followed the need of storing property. But there was great variety in human character. One family would store more than enough, through fear of possible accidents; another barely sufficient, another not enough. When this last family came to be in want, and needed to be fed, what was to be done? Clearly it would not be right that this unthrifty family should be fed by the thrifty; that would encourage waste and extravagance, and even idleness. The inevitable result was that the unthrifty family came to the thrifty, and gave its labour for sustenance, and so they had at once the distinction between capital and labour. All capital, as capital, was the result of abstinence, or thrift, if they liked to call it so; all property, as such, was the result of labour.

Domestic life was an initial need of humanity, the seed of all that was most excellent, and the moral basis of private property. Some might rise to even a higher level than this conception of domestic life, but not the great body. With the great body, domestic life was the source of all that was best, and it was consecrated by the title of relationship—Father—in which Christ revealed God to men. In the same way our Lord showed the relation of husband and wife to be consecrated, by declaring that this is the relation between Himself and His Church, the great Christian body.

